# COVID-19 Infection and Acute Pulmonary Embolism in an Adolescent Female With Sickle Cell Disease

**DOI:** 10.7759/cureus.12348

**Published:** 2020-12-28

**Authors:** Sushma Kasinathan, Hasina Mohammad Ashraf, Sheera Minkowitz, Adebayo Adeyinka, Keneisha Bailey-Correa

**Affiliations:** 1 Pediatrics, The Brooklyn Hospital Center, Brooklyn, USA

**Keywords:** sickle cell disease, pulmonary embolism, covid-19, sickle cell hbsc

## Abstract

A previously healthy 20-year-old female presented to the emergency room in April 2020 with complaints of shortness of breath, chest pain, and cough. She was diagnosed with coronavirus disease 2019 (COVID-19) infection and pulmonary embolism (PE). Workup for anemia led to the diagnosis of sickle cell disease (SCD). Patients diagnosed with COVID-19 are at an increased risk for the development of PE and venous thromboembolism (VTE). Anticoagulation prophylaxis and escalation to treatment dosing are recommended in patients admitted with moderate to severe symptoms of COVID-19. PE and VTE are relatively uncommon in the pediatric and adolescent population. Most commonly, patients are diagnosed with thrombophilia or have an underlying hypercoagulable state such as with SCD. Also, symptoms of COVID-19 infection, acute chest syndrome (ACS), and PE can have overlapping features. In this report, we present a case of a late adolescent female with SCD, who was diagnosed with COVID-19, and whose condition was complicated with PE.

## Introduction

Originating in the Wuhan province of China and spreading quickly to the rest of the world, coronavirus disease 2019 (COVID-19), which is caused by the severe acute respiratory syndrome coronavirus 2 (SARS-CoV-2) virus, has become a global pandemic [1]. The COVID-19 infection presents differently in the adult and elderly population compared to the pediatric population. This holds true in the late adolescent period as well, which is defined by the American Academy of Pediatrics (AAP) as the ages of 18-21 years. The infected patients have been shown to have an elevation of pro-inflammatory markers [1]. Recent reports have shown that the virus can cause an increase in hypercoagulability with multiple cases of pulmonary emboli noted in adults [1]. There have been several groups of people, such as the elderly, that are especially at risk of developing complications. The case we present is that of a previously healthy adolescent who contracted SARS-CoV-2 and was found to have sickle cell disease (SCD). SCD is a group of inherited hemoglobinopathies where there are mutations of the beta-globin chain resulting in sickled hemoglobin in the body [2]. As per the Centers for Disease Control and Prevention (CDC), SCD affects about 100,000 people in the United States. It is especially prevalent in the African American and Indo-Caribbean population. One of the many complications of this disease includes a chronic hypercoagulable state, which can increase the risk of thromboembolic events [2]. This hypercoagulable state complicated the disease course of our patient, leading to the development of pulmonary emboli [3,4].

## Case presentation

**Figure 1:**
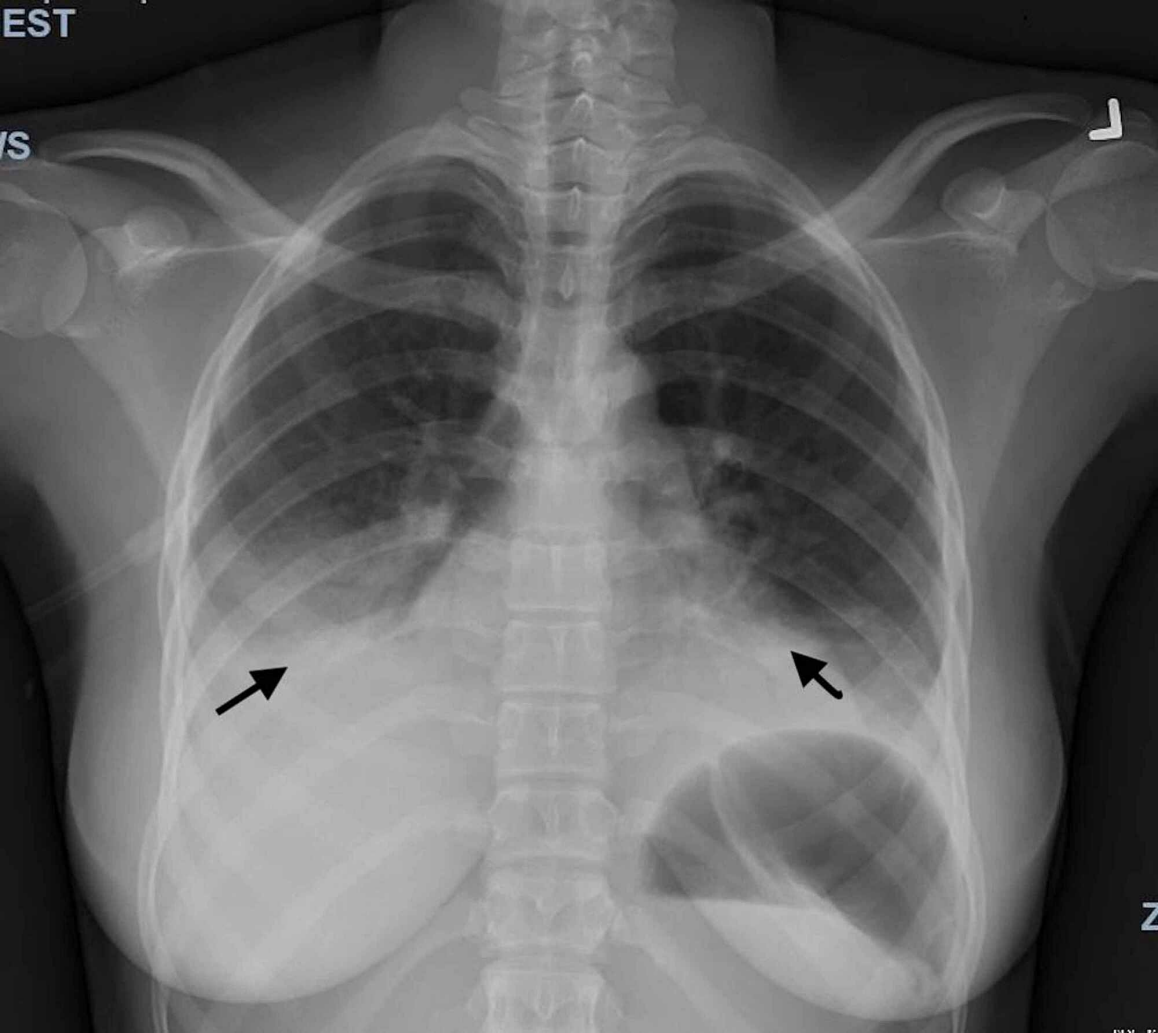
Chest X-ray (PA and lateral) The image shows a further increase in the ground-glass opacities within both lungs with increased opacity within the retrocardiac region with air bronchogram. Worsening airspace disease indicative of pneumonia is also observed PA: posteroanterior

A 20-year-old female, who had immigrated to the United States from Ghana five years ago, with no previous known medical history, presented to the emergency department (ED) with a productive cough, shortness of breath, and chest pain for three days. She also reported new-onset back pain for one day. The chest pain was in the mid-sternal region, non-radiating, and pleuritic. The patient had visited the ED a day earlier for similar complaints and undergone a chest X-ray (CXR), which showed an infiltrate of the right lower lobe and ground-glass opacities involving the left lower lobe. On presentation, she was tachycardic with a heart rate of 133 bpm, tachypneic with a respiratory rate of 24, and blood pressure of 130/73 mmHg. However, she was afebrile with a temperature of 99.1 °F. She initially had a low oxygen saturation in the mid-80s, which improved to mid-90s once she was placed on 2L of oxygen via nasal cannula. The physical examination was remarkable for labored respirations at rest, accessory muscle use, and diffusely decreased breath sounds bilaterally. A repeat CXR showed worsening of the airspace disease in both lower lobes, with an increase in the ground-glass opacities bilaterally (Figure 1). Initial labs from the ED and day one are summarized below in Table 1.

**Figure 2:**
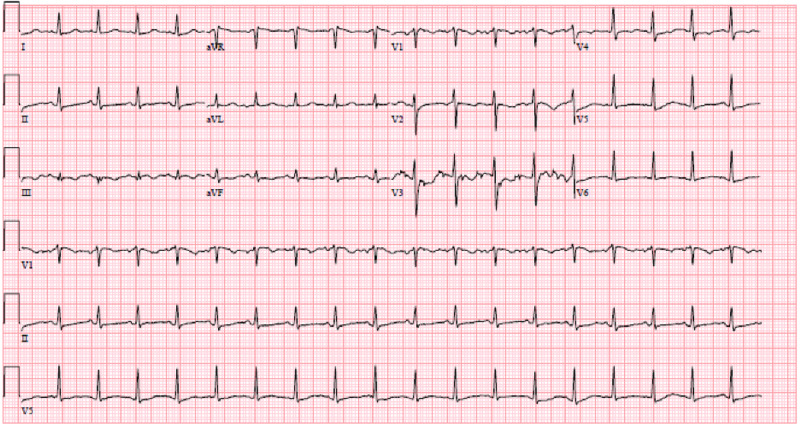
EKG showing sinus tachycardia EKG: electrocardiogram

**Figure 3:**
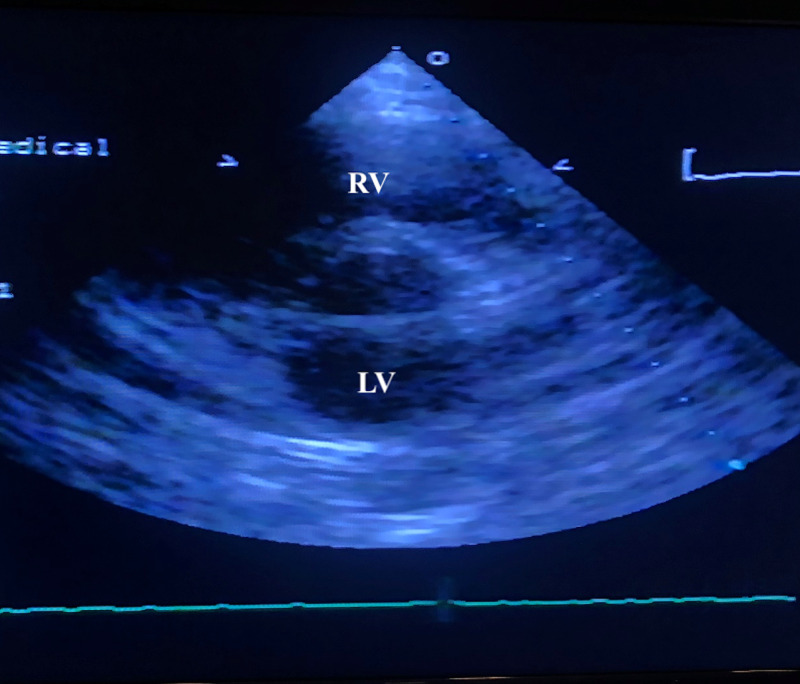
Echocardiogram Parasternal short-axis view showing normal right ventricle and left ventricle

**Figure 4:**
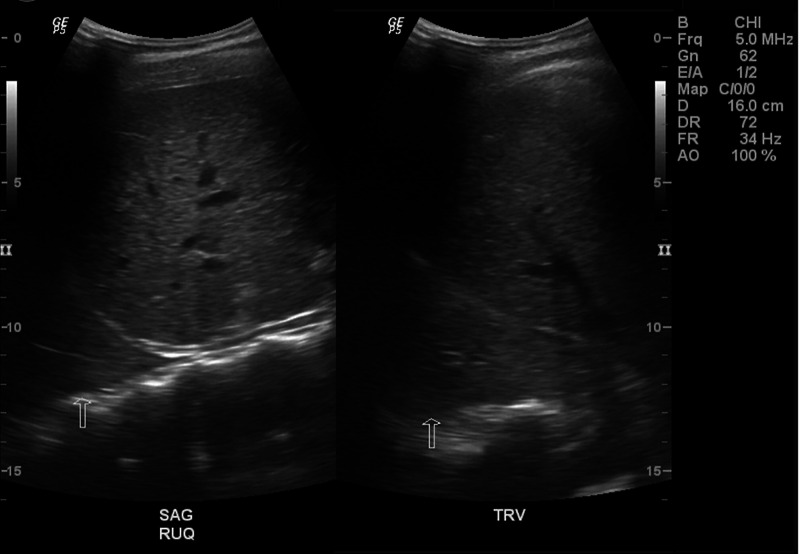
Ultrasound chest There are small bilateral pleural effusions. The fluid appears to be complex

**Figure 5:**
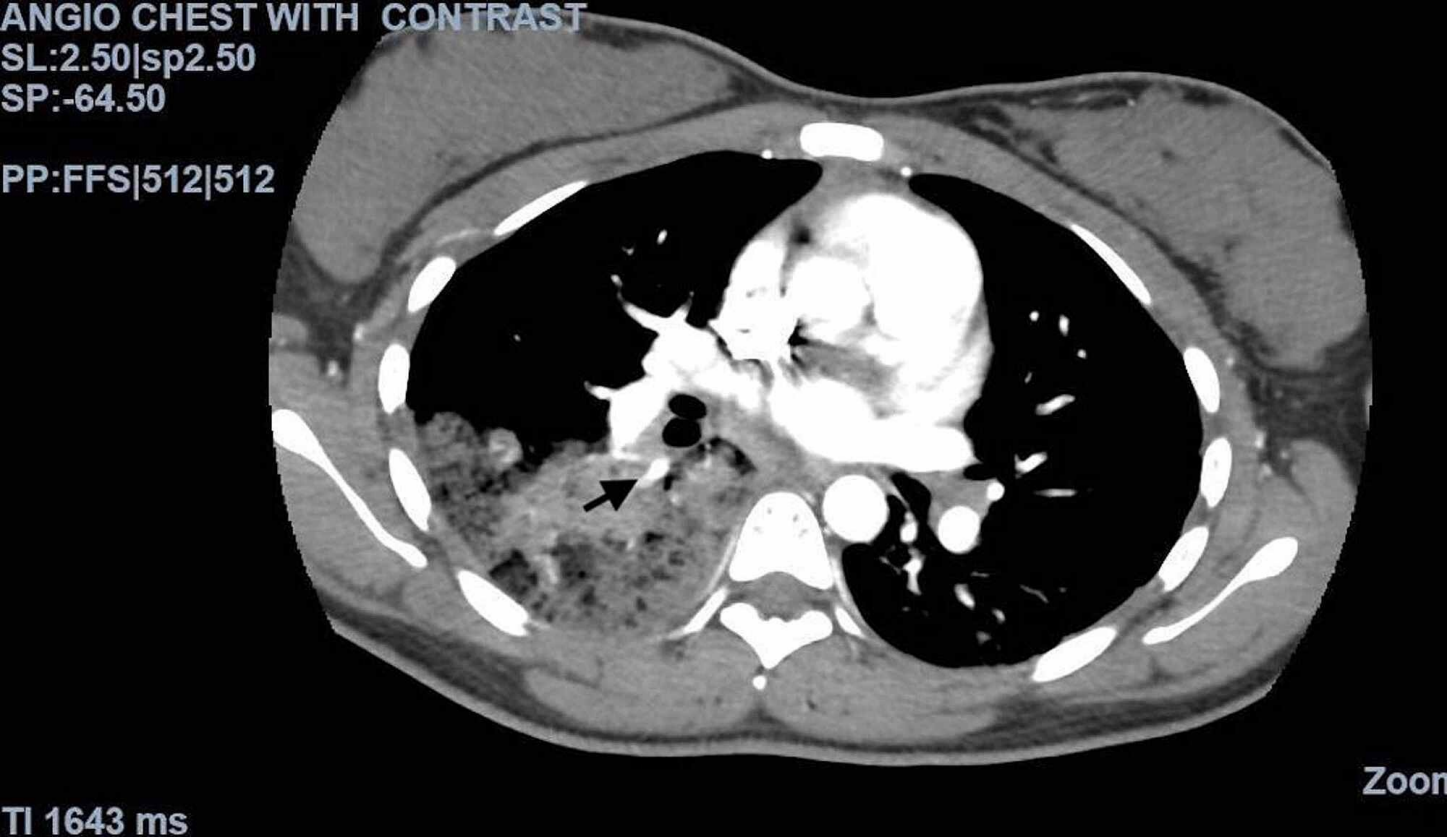
CT angiogram of the chest with IV contrast The image shows acute pulmonary emboli involving the right lower lobe CT: computed tomography; IV: intravenous

**Figure 6:**
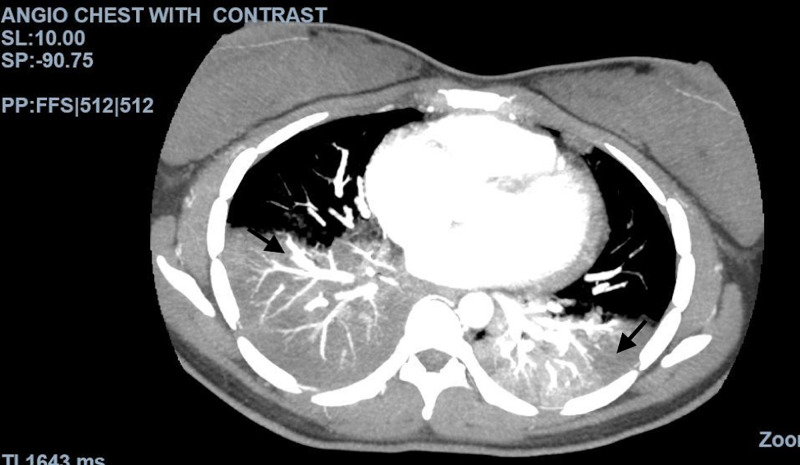
CT angiogram of the chest with contrast The image shows multifocal consolidation predominately involving the lower lobes, characteristic of pneumonia seen in COVID-19 CT: computed tomography; COVID-19: coronavirus disease 2019

On day two of hospitalization, she was found to be SARS-CoV-2 polymerase chain reaction (PCR)-positive. Blood culture showed no growth after five days. Since there was a mild elevation of troponin, an electrocardiogram (EKG) was performed, which showed sinus tachycardia (Figure 2). Troponin continued to remain elevated reaching a maximum level of 0.40 ng/ml. An echocardiogram showed a structurally healthy heart (Figure 3). Due to possible pleural effusions observed on CXR, an ultrasound of the chest was obtained, which confirmed small-sized bilateral pleural effusions (Figure 4). A CT angiogram was performed, which showed bilateral pulmonary emboli, predominately in the right lower lobe (Figure 5) with multifocal consolidation involving both lobes (Figure 6). The patient was admitted to the pediatric intensive care unit (PICU), and started on intravenous fluids and high flow nasal cannula (HFNC). The patient was also started on a course of ceftriaxone 2 grams daily and a five-day course of azithromycin for presumed pneumonitis. The patient continued to have a fever with worsening back pain. Oral analgesics were inadequate to control the back pain, and hence she was escalated to intravenous opioids, which alleviated her symptoms. A course of hydroxychloroquine 400 mg twice daily for three days and then 400 mg nightly for a total of seven days was initiated. The patient was also started on enoxaparin 1 mg/kg/dose subcutaneously twice daily for her bilateral pulmonary embolism (PE). A rheumatologic workup was done, which showed elevated cardiolipin immunoglobulin G and immunoglobulin M levels (Table 1). More significantly, the results of hemoglobin electrophoresis performed were consistent with hemoglobin SC disease (Table 2). The hematology/oncology team was consulted, who recommended packed RBC transfusion that resulted in an overall improvement in her respiratory status. In the subsequent days, the patient's fever resolved, and she was successfully weaned off HFNC to room air. She was discharged home on enoxaparin subcutaneously for at least three months with appropriate follow-up appointments.

**Table 1:** Laboratory values during the hospital stay ED: emergency department; PICU: pediatric intensive care unit; ESR: erythrocyte sedimentation rate; CRP: C-reactive protein; BNP: brain natriuretic peptide; AST: aspartate aminotransferase; ALT: alanine transaminase; LDH: lactate dehydrogenase; PT: prothrombin time; PTT: partial thromboplastin time; INR: international normalized ratio; IgM: immunoglobulin M; IgG: immunoglobulin G

Parameter	Reference value	ED	PICU Day 1	Day 2	Day 3	Day 4	Day 5	Day 6	Day 7	Day 8	Day 9	Day 10
White blood cell count	4.8-10.8 x 10^3^/cmm	33.3	33.2	43.7			33.2	31.8	24.9	21.2	17.9	15.5
Hemoglobin	11.5-14.1 g/dL	8.5	8.3	8.7			7.9	8.3	6.5	9.5	8.1	10.4
Hematocrit	35-42%	26	25	26			25	24	19	27	24	31
Platelet count	130-400 K/cmm	491	461	586			672	711	631	637	563	729
Neutrophil %	42.2-75.2%	81.7	90.7	88.5			64.6	84	86.8	63.6	79.6	40.9
Lymphocytes %	20-51%	2.8	1.0	0.9			4.3	5	3.5	6.4	4.2	52.9
Monocytes %	1.7-9.3%	6.9	5.6	8.6			26.8	8	7.4	25.6	13.6	0.5
ESR	0-20 mm/hr	43							90	84	120	
CRP	<5 mg/L	239.70		170.65			132.29		187.33	164.03	90.44	
Ferritin	10-204 ng/ml	1,360							1,528			
Creatinine Kinase	29-168 U/L	57										
Troponin	<0.03 ng/ml	0.21	0.26	0.35	0.29	0.29						
BNP	<100 pg/ml				21							
Lactic acid	0.5-2.2 mmol/L	2.1										
Sodium	136-145 mmol/L	135	137	141			135			136		
Potassium	3.5-5.1 mmol/L	3.7	3.8	4.0			4.6			3.9		
Chloride	98-107 mmol/L	99	99	102			101			102		
Bicarbonate	22-29 mmol/L	24	27	26			22			27		
AST	8-34 U/L	31		28			51			25		
ALT	6-55 U/L	12		18			34			27		
LDH	125-220 U/L	561					677					
PT	9.5-11.4 sec								12.2			
PTT	20.6-29.3 sec								22.1			
INR	0.9-1.1								1.1			
Haptoglobin	14-250 mg/dl								394	389		
D-dimer	<0.5 mg/L	>4.40							>4.40			
Cardiolipin IgM	<12 gpl			33								
Cardiolipin IgG	<14 gpl			19								

**Table 2:** Hemoglobin electrophoresis

Parameter	Percentage	Reference value
Hemoglobin F	8.0% (H)	(0-2.0%)
Hemoglobin S	47.7% (H)	ABS%
Other hemoglobin	Hemoglobin C: 44.3%	
Interpretation	Consistent with hemoglobin SC disease	

## Discussion

We presented an unusual case of PE in a late adolescent patient. PE and venous thromboembolism (VTE) have been reported to be known complications of COVID-19 infections [1]. Multiple cases of PE have been reported in patients with poor or worsening respiratory status in the adult population [3]. Patients often present with tachypnea, dyspnea, hypotension, and elevated D-dimer and fibrinogen [3].

PE is a rare occurrence in the pediatric population. In general, pediatric patients have fewer comorbidities and risk factors for PE compared with the adult population. Annually, PE occurs in 0.86 of 10,000 children in the general population [3]. The risk factors for developing VTE include recent surgery, trauma, obesity, prolonged immobilization, initiation of hormone therapy, active cancer, and cigarette smoking [3]. Of the many genetic risk factors, the common ones include Factor V Leiden mutation and prothrombin gene mutation. Our patient was noted to have elevated cardiolipin antibodies that can occur in the setting of viral infections such as COVID-19 [4].

SCD is another element of this case that was unknown at the time of our patient’s initial presentation. The patient did not have a newborn screening in her home country of Ghana. Hemoglobin electrophoresis was performed as part of the workup for anemia. Our patient was found to have hemoglobin SC disease, a variant resulting from a different point mutation in the globin chain [5]. Acute chest syndrome (ACS) is a well-known complication of SCD, which involves fever with or without new respiratory symptoms and the presence of new infiltrates on CXR [6]. This can be triggered by viral infections such as COVID-19. The clinical picture for both ACS and COVID-19 can be similar [7]. Management for both includes giving intravenous fluids and providing antibiotic coverage with ceftriaxone and azithromycin for atypical pneumonia [8]. Simple blood transfusions can also be given. In our patient, we saw that these blood transfusions helped her overall respiratory status.

Patients with SCD are at an increased risk of developing thromboembolic diseases [5]. Fat embolism syndrome marked with abnormal coagulation profiles used in fat embolism scoring indices is an early warning sign of fulminant deterioration [9]. The pathophysiology of sickle cell-associated thromboembolic events is unclear but it is speculated that sickled RBCs increase the production of thrombin and its complexes, thereby increasing the hypercoagulability [10]. Many studies suggest that there is a hemostatic imbalance associated with a decrease in anticoagulant proteins, an increase in platelet activation factors, leading to increased activation of the coagulation cascade [11]. Patients with increased risk factors for venous stasis, endothelial injury, or hypercoagulability, comprising the Virchow's triad, should be on prophylaxis with low molecular weight heparin [12]. Once thromboembolism is speculated to be the diagnosis or when confirmed with contrast-enhanced CT angiogram, anticoagulation treatment should be initiated [13]. We treated our patient with enoxaparin with a favorable outcome.

## Conclusions

Pulmonary emboli are seldom seen in the pediatric population. When they do occur, it is essential to investigate the underlying causes. Due to the association of COVID-19 infection with PE, a high index of suspicion is necessary for the diagnosis of PE, since on many occasions, imaging is not feasible. Prophylactic anticoagulation of all hospitalized COVID-19 patients has been suggested with escalation to therapeutic dosing when a PE is suspected. As noted in the setting of COVID-19, further consideration should be given to patients with other comorbidities such as SCD.
